# Extra-Limites

**Published:** 1825-10-01

**Authors:** 


### XIII.

### EXTRA-LIMITES.

### I.

*Dr. Gordon Smith to the Editor.*

Dear Sir,—You will oblige me by inserting in the next Number of your Journal, the following observations, suggested by the manner in which your Reviewer of my work, on Medical Evidence, has quoted and animadverted on a passage contained in that book. My desire is to treat the matter amicably and dispassionately; while at the same time justice to the Profession as well as myself, requires that I should submit something in the way of explanation.

The passage in question is brought forward in pp. 175 and 176; and standing, as it there does, carries an import which I little expected to recognise in it, and one which I never intended it to bear. This arises partly from its being separated from the context; but, in no small degree, perhaps, from unfair typography. In the original there are but three words printed in a distinguished manner, and these it was almost imperative so to print—whereas, in the Journal, there are others exhibited, in the same way by which their "*sarcastic*" tendency is somewhat strained.

I am quite aware that it would not become me to answer the charge against those who have been occupied with the cultivation of Forensic Medicine. The study is a new, and hitherto, unprofitable burden; for which some may consider that they owe us no thanks; but it may afford a ray of consolation to reflect that opposition has generally waited even on those who have become acknowledged benefactors to science: of this I have no doubt that, whenever there shall be more "fees" to be got in Westminster Hall, than in the sick-room, Forensic Medicine will be considered a most useful as well as important study, and medico-legal knowledge will not lack cultivation. The gravamen however of the present case is, that I have contributed to throw discredit on a branch of medicine, extensively cultivated and highly esteemed—in doing which, I have laid myself open to an imputation of illiberality. I shall not enter farther into the merits of the question than can be done by a brief elucidation of the particular point at issue. The subject at large is one that would require much greater scope than can here, on either side, be afforded; but it is one into which I certainly should not shrink from going, were it required of me. At the same time I would remark, that the point actually calling for notice is not the importance or utility of midwifery itself, for that I maintain to be not unfairly treated even in the passage alluded to, but an abuse which belongs to that study in common with several others, composing the aggregate of medical education.

It is true that I have exemplified this abuse by quoting a practice on the part of too many accoucheurs, which, if generally followed, must destroy the efficacy of the medical schools, as the only sources to which we have to look for a continued supply of able and reputable practitioners, in whatever department; but I have brought no charge against either midwifery or its cultivators. I hesitate, not, however, to repeat concerning that study as well as others, (which might also have been selected by way of example,) that among fifty persons well versed in its science and competent to exercise the art, there may not be one qualified to set up as a public professor, and announce himself as the head or founder of a school. I put forward this statement, without fear of offending, much less of injuring either those learned and talented individuals who are deservedly eminent as Professors of Midwifery, or the important claims of their particular province.

To introduce, among observations connected with a work professedly on *evidence*, a mere assertion, instead of a proof, would be somewhat inconsistent—otherwise I might restrict myself *to say* that I do not despise midwifery—but I may as well inform my Reviewer that the study of midwifery, *to the fullest extent*, entered among my original qualifications to become a physician, and has ever since occupied a fair portion of my leisure hours. This being the case, it is for the best judges to draw the fair inference as to my estimation of its importance and utility. It is true I do not practise as an *accoucheur*; nor do I speak French in preference to English.

I would further observe that, in the regret I was led to express at the determination of the University of Edinburgh to exact attention to midwifery, (a circumstance which I believe the general voluntary practice of the students, in that School, hardly required to be made imperative,) with a view to obtaining academical honours, *in preference* to legal medicine, I merely lamented the omission of the latter. I am not unprepared to maintain, (though this be not the place,) both the importance and magnificence of Public Medicine, and, perhaps, to prove its superiority, even to midwifery, in the *due* sense of the word—but that *problem* I waive for the present. That the omission in question was considered rather glaring, is proved by its having been subsequently rectified.

And now, by way of conclusion, allow me to say, that, while, on the one hand, I entertain all becoming, and, I might add, all desirable respect for midwifery, and while I fully acknowledge the "ability, intelligence, and usefulness of physician-accoucheurs," I can discriminate between that which is entitled to praise and that which is not—that which is reality, and that which is pretension—and I know that if all physicians are not accoucheurs, too many of these are not physicians. It may not be amiss to hint to my friend the Reviewer, (whose ideas of the main chance are sufficiently comprehensive to encourage even the student of "forensic subtelties,") that the ready, though laborious mode of getting "fees," afforded by midwifery, might not be a very insufficient reason for its extensive modern cultivation.

I remain,

*Cotmandene, Surry.* Yours truly,

July 15, 1825. J. G. SMITH.

### II.

### DR. DAVIS ON THE FORCEPS.

Mr. Weiss, the Surgeons' Instrument Maker, having in the last Number of the Medico-chirurgical Review, charged the author of the Elements of Operative Midwifery, recently published, with having adopted an invention of his (Mr. Weiss) without acknowledging it, Dr. Davis requests permission, in this number of the Journal, to give a short statement of the actual facts of the case.

Mr. Weiss was employed by Dr. Davis to make for him a pair of forceps with blades of unequal lengths for the purpose represented in plate X. of the Elements of Operative Midwifery.

Dr. Davis gave ample instructions as to the joint at (b) which he required to be made in the blade of the longer counterpart of his forceps; and that there might be no excuse for failure or inaccuracy in the construction of any part of the instrument, he furnished Mr. Weiss with a pelvis and an artificial child, which he accompanied with directions by demonstration on these models, as to the special properties of the new forceps, more precise and intelligible than it was in his power to have conveyed by any drawing. Mr. Weiss, at this period, had not only not invented his jointed lever, but had never seen nor heard of a vectis with a jointed blade, till Dr. Davis told him of the living lever of Dr. Aitkin. Mr. Weiss's vectis was not constructed for many weeks after he had executed Dr. Davis's order: moreover this vectis, now represented as the original instrument, was made to the order of a gentleman from the country, who took a fancy to the principle of it, in consequence of Dr. Davis's forceps having been shewn to him by Mr. Weiss

We understand that Mr. Robert Hicks, Surgeon, of Conduit-Street, has invented a very ingenious Bath of a most portable nature, and possessing the greatest facilities of application, for which he has taken out a patent. We shall be able to state more particulars in our next.

### III.

At the Annual General Meeting of the Associated Apothecaries and Surgeon-Apothecaries of England and Wales, held by public advertisement, at the Crown and Anchor Tavern, Strand, July 6th, 1825,

JOSEPH HAYES, Esq. President,

The General Committee presented the following Report.

In conformity with established usage, your Committee has the honor to lay before the General Meeting a Report of its proceedings during the last year.

Anxious to effectuate the important objects for which it was appointed, your Committee has deliberated, at various times, on the best means of promoting the public welfare, and ameliorating the condition of the general practitioner, by improving his education, and increasing his attainments.

The imperfections and disadvantages of the Act of 1815, commonly called the Apothecaries' Act, have been so often alluded to, that it is unnecessary on this occasion to enter into any detail of them.

Since the last General Meeting, a Bill has been brought into Parliament, at the instance of the Society of Apothecaries, to amend the Act of 1815, but your Committee has to regret that, previously to its introduction into the Lower House, no opportunity was offered your Committee of proposing those ameliorations which the public good and justice to the general practitioner alike require; neither was any intimation given, unless a verbal communication avowedly unauthorized may be so termed, of the objects to be obtained by the proposed alteration; thus circumstanced your Committee felt it their duty to seek, through proper channels, precise information, and to endeavour to obtain, by representation to the legislature, those alterations which should conduce to the public benefit, and to the usefulness and respectability of the medical practitioner. A Sub-committee was accordingly appointed to carry these intentions into effect, the gentlemen composing it had an interview with Mr. Brougham, by whom they were received very courteously, and subsequently they conferred with Mr Hume, who entered with them into a patient and comprehensive investigation, both of the existing Act, and of the Bill for its amendment.

The enlarged view with which this distinguished legislator took of the subject, tracing its various bearings as relating both to the community and the profession, fully prepared them to expect from him that distinguished zeal and talent which he has since evinced in his place in Parliament, and which entitle him to the warmest thanks, not only of this Association, but also of every member of the medical community.

The Act, however, possesses so many inherent imperfections, that notwithstanding the proposed amendments, which, however, will be found to be important meliorations, it is to be feared it will still prove very inadequate to the more enlightened purposes originally contemplated by this Society.

The rapid progress of knowledge, the increasing attention of the legislature to the wants of the community, and above all, the intended establishment of a Metropolitan University, afford a just expectation that the period is not far distant when the ardent hopes of the Association, as subservient to the best interests of mankind, will be realized.

*Published by Order of the General Meeting,*

JOHN POWELL, *Secretary.*

